# Comment on "Heart Injury with Projectile Lodged Inside the Heart"

**DOI:** 10.21470/1678-9741-2019-0601

**Published:** 2019

**Authors:** Noedir Antônio Groppo Stolf

**Affiliations:** 1 Instituto do Coração do Hospital das Clínicas da Faculdade de Medicina da Universidade de São Paulo (InCor-HCFMUSP), São Paulo, SP, Brazil.

I read with much interest the article of Volpe et al.^[1]^ on "Heart injury with projectile lodged inside the heart". When consulting the references, we found three important articles by Brazilian authors.

Nevertheless, they missed two articles that we published in 1987^[2]^ and 1988^[3]^ . The first one reports one case and the second one describes two cases and reviews the literature^[3]^. The first patient underwent surgery on January 1985, on an emergency basis, at another institution and was then referred to us; the second one was directly referred to us on May 1985. The bullet was in the interventricular septum (IVS) in the first case, projecting in the left ventricle outflow track, and the second patient was sent to the operating room after a rapid blood infusion. The bullet localization in the second case was performed by an epicardial echocardiogram.

In both cases, under cardiopulmonary bypass and cardioplegic arrest, the right atrium was opened, the bullet palpated, an incision in the right side of the septum was made, the bullet removed and the incision of IVS closed with pledged stitches.

So, I believe that these cases are interesting, worth of mention and the surgical approach was simpler than the one used by Volpe et al.^[1]^.
